# Shear Wave Elastography in the Diagnosis of Hand Tumours

**DOI:** 10.1155/2019/2736529

**Published:** 2019-02-24

**Authors:** Debora Schivo, Ergys Gjika, Aurélien Traverso, Sébastien Durand

**Affiliations:** ^1^Department of Plastic, Reconstructive and Hand Surgery, University Hospital of Lausanne, CHUV, Lausanne, Switzerland; ^2^Department of Hand Surgery, University Hospital of Geneva, HUG, Genève, Switzerland; ^3^Department of Orthopaedics and Traumatology, University Hospital of Lausanne, CHUV, Lausanne, Switzerland

## Abstract

Diagnosis of hand tumours by conventional imaging remains difficult. Shear wave elastography (SWE) is a noninvasive method used to quantitatively assess the mechanical properties of tissues. We provide the first report of “histoelastographic” data concerning a finger tumour. Our data support the notion of ultrasound assessment using multiple parameters including morphology, elasticity, viscosity, and microflow vascularization likely contributing towards a more precise diagnosis in the future.

## 1. Introduction

Tumours of the hand and wrist represent 12.8% of soft tissue lesions [1]. There remains no noninvasive solution to establish with certitude the diagnosis of musculoskeletal tumours and of the hand in specific [2]. Standard radiography reveals aspecific signs such as cortical condensation, erosion, or periosteal reaction. While B-mode ultrasound imaging can be used to classify soft tissue lesions as cystic or solid depending on their echogenicity [3], the use of ultrasound and/or MRI is insufficient. Histological examination of a targeted biopsy is considered to be the best method to provide a definitive diagnosis of a suspect lesion [2] but an error rate of 17.8% has been found [4], which significantly reduces their utility. The surveillance of certain recurrent tumours is currently poorly evaluated by simple morphological examination. Furthermore, progress towards less invasive procedures in the treatment of certain tumours imposes a precise diagnosis as prerequisite to any intervention [5–8].

All tissues, whether healthy or pathological, present specific biomechanical properties relating to their specific architecture and which can be expressed using stress/strain curves or Young's modulus. One could reasonably assume therefore that knowledge of the elastic properties of different tissues might be indicative of their nature.

Shear wave elastography (SWE) is a recent quantitative technique for assessing the elasticity of soft tissues. It uses an acoustic radiation force impulse (ARFI) generated by a focused ultrasound beam and quantifies the shear wave velocity (in meters per second) and stiffness (Young's modulus in kilopascal) of tissues [9]. Initially used in the early nineties in vitro [10], its use has since then progressively increased within clinical exploration as a diagnosis and sometimes prognostic tool examining breast, liver, thyroid, prostate, and musculoskeletal pathologies [7, 11–13]. SWE has been shown to provide quantitative and reproducible information on solid breast lesions and serve as an accurate diagnostic tool for discriminating malignant and benign lesions, thereby avoiding biopsy [14].

Elastography has been used to assess upper limb musculoskeletal tissue and seems to be interesting in the diagnosis of many pathologies: lateral epicondylitis [15], rotator cuff tendon pathology [16], trigger finger [17], carpal tunnel syndrome [18], upper limb tendon transfer [9], and finger pulp reconstruction [19]. The aim of this case is to demonstrate the technical feasibility of SWE for evaluating Young's modulus of infracentimetric tumours of the hand, which commonly occurs in hand surgery.

## 2. Case Report

A 45-year-old woman without any history of trauma presented with a painful and hard mass located within her right thumb pulp. The tumour showed adherence to deep tissues but not signs of local inflammation. Standard radiographs were unremarkable. The ultrasound examination of the tumour reported an encapsulated mass with regular and well-defined margins.

**Figure 1:**
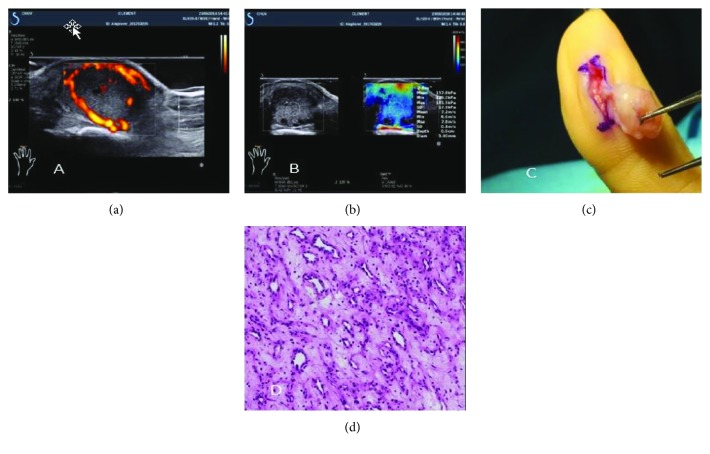
(a) Doppler ultrasound angio PL.U.S mode. Peripheral ring-like vascularization of the mass. (b) SWE using a high-frequency probe (SHL 15-4, average frequency 12 kHz). (c) Surgical excision of the tumour. (d) Histology: lobular capillary haemangioma.

Doppler ultrasound using angio PL.U.S mode showed a homogenous ovoid mass with peripheral ring-like vascularization (Figure 1(a)).

SWE performed (Aixplorer®, Aix-en-Provence, France) using a high-frequency probe (SHL 15-4, average frequency 12 MHz) centred with a quantitative Q-box, with a circular region of interest of 3 mm diameter, on the mass revealed shear wave speed and modulus of elasticity of, respectively, 7.2 (6.6-7.8) m/s and 157.8 (129-181.5) kPa in the transverse plane and 5 (4.6-5.7) m/s and 75.8 (63.1-96.4) kPa in the sagittal plane (Figure 1(b)). The Q-box circle has a maximal precision of 1 mm; therefore, very small tumours can be detected and analysed.

Surgical excision of the tumour (Figure 1(c)) was carried out and the histopathological examination revealed a deep lobular capillary haemangioma (Figure 1(d)). The postoperative follow-up was without complications.

## 3. Discussion

Elastography is an imaging modality which maps the elastic properties and stiffness of soft tissue by different techniques. Strain elastography allows qualitative analysis, based on the deformation of the tissues (strain) for a given stress, and results in a qualitative map of the elastic modulus distribution, defined as an elastogram. While true quantitative measurements cannot be taken from this elastogram, a semiquantitative evaluation can be determined with the strain ratio, which represents an index of the relative elasticity between a chosen region of interest (ROI) and the surrounding tissues [20]. SWE does however provide quantitative information relating to tissue elasticity and is more reproducible than strain elastography owing to the standardised applied stress [21].

Studies into the use of SWE to investigate musculoskeletal tumours or masses are relatively few in number. Most previous studies on the use of sonoelastography for differentiating benign from malignant lesions adopted qualitative scales [22–26] (Table 1).

Different process models have been used to develop the SWE technique. The reproducibility, based on the results obtained using the different models, is unclear. The literature demonstrates certain discrepancies among the elastographic data obtained with different software [27]. We also found heterogeneity among the studies we analysed in terms of the probes used, with frequencies between 5 and 40 MHz (Table 1).

The data in the literature suggest that shear wave velocity measurements are reproducible and that malignant masses may have slower shear wave velocities than benign masses [28, 29].

To our knowledge, only three published studies concerned with the application of SWE in assessing musculoskeletal soft tissue masses presented quantitative elastographic results [20, 28, 29].

Using SWE to analyse soft tissue tumours, Pass et al. [28] reported an average 30% slower longitudinal shear wave velocity of malignant masses as compared to benign masses. The authors concluded upon this finding representing some evidence of an association between lower shear wave velocities and malignancy. Taljanovic et al. [20] described the applications of SWE in the evaluation of various pathologic conditions of the musculoskeletal system and its utility in the characterization of soft tissue masses.

Larger prospective studies will be needed to establish the diagnostic value of SWE in musculoskeletal tumours. While B-mode and Doppler imaging provide information on acoustic impedance and vascular flow allowing a more detailed interpretation of the microvasculature inside a tissue lesion, SWE provides information about the tissue stiffness and could be a useful complementary tool.

Ultrasound imaging has been evolving towards multiparameter assessment of soft tissue tumours and different techniques have now been developed to allow their more detailed analysis. SWE has been shown to have potential as a diagnostic and therapeutic tool not only for diseases of the breast, liver, thyroid, and prostate but also for musculoskeletal pathologies and in particular those of the hand [9, 17–19].

Ultrasound elastography appears to be a good complementary tool used in conjunction with B-mode ultrasound. This technique is reliable and reproducible and can be used and further developed to reinforce the assessment and enhance diagnostic confidence in malignant lesions of the musculoskeletal system [24, 25, 28, 29].

In the future, we foresee the completion of hand tumour assessment with a measurement of tissue viscosity, which has already proven to be useful in the assessment of liver lesions.

In our centre, we are developing a database of “histoelastographic” data on soft tissue tumours of the hand obtained using ultrasound.

## Figures and Tables

**Table 1:** Elastographic data for musculoskeletal tumours in the literature.

Article	Year	ROI	System	Technique	No.	Mass	Quantitative		Qualitative
Pass et al. [29]	2017	Dim. 2 × 2 mm VR 0-10 m/s	Acuson S3000 (9-4 MHz)	SWE (ARFI)	105	S	Long. 2.94 m/s	Trans. 2.93 m/s	Red
						H	Long. 2.57 m/s	Trans. 2.56 m/s	Blue

Taljanovic et al. [20]	2017	SW-velocity VR 0.5-15 m/s	Acuson S3000 (9-4 MHz)	SWE	7	S	Lipoma: 1.74-5.52 m/s Tophus: 7.32 m/s Fibroma: 5.93 m/s Epidermoid cyst: 2.76 m/s Baker cyst: 2.8 m/s Enchondroma: 9.21-15 m/s		Blue
						H	Osteosarcoma: 4.12 m/s		Red

Pass et al. [28]	2016	Dim. 6 × 7 mm VR 0-6 m/s	Acuson S2000 (9-4 MHz)	SWE (ARFI)	50	S	Long. 1.36 m/s	Trans. 1.92 m/s	Red
						H	Long. 2.17 m/s	Trans. 2.15 m/s	Blue

Hahn et al. [22]	2017	ROI A/B (A: lesion; B: adjacent area)	Acuson S2000 (5.5-18 MHz)	Strain elastography Strain ratio Elasticity score	73	S	/		SR: 1.03 ± 0.93 ES: 3.08 ± 1.44 Red
						H	/		SR: 0.49 ± 0.45 ES: 3.76 ± 0.97 Blue

Park et al. [23]	2015	ROI lesion	LOGIQ E9 (6-15 MHz) IU22 (5-12 MHz)	Strain elastography score (1-4)	103	S	/		Score 1-2 Red
						H	/		Score 3-4 Blue

Magarelli et al. [24]	2014	ROI lesion	My Lab 70 XVG (5-12 MHz)	Strain elastography score (1-5)	32	S	/		Score 1-3 Red
						H	/		Score 4-5 Blue

Lee et al. [25]	2014	ROI B/A	Acuson S2000 (5.5-18 MHz)	Strain elastography	34	S	/		Lipoma (19): SR 0.83 ± 0.18 Ganglia (6): SR 2.78 ± 0.48 Epiderm. cyst (5): SR 0.17 ± 0.21 Pilomatricoma (4): SR 0.13 ± 0.02 Blue
						H	/		Red

Lalitha et al. [26]	2011	ROI lesion	GE E8 (8-12 MHz)	Real-time compression elastography	Few	S	/		Haemangioma Ganglion cyst Lipoma Red
						H	/		Blue

S: soft; H: hard; SR: strain ratio; ES: elasticity score; ROI: region of interest; Long.: longitudinal; Trans.: transversal.
